# Integrated Thermofluid Lumped Parameter Model for Analyzing Hemodynamics in Human Fatigue State

**DOI:** 10.3390/bioengineering10030368

**Published:** 2023-03-17

**Authors:** Xiaoling Ding, Ying He, Youqiang Chen, Yueping Wang, Lili Long

**Affiliations:** Key Laboratory of Ocean Energy Utilization and Energy Conservation of Ministry of Education, School of Energy and Power Engineering, Dalian University of Technology, Dalian 116024, China

**Keywords:** fatigue, cardiovascular system, heat transfer, lumped parameter, peripheral resistances

## Abstract

It is well known that driving while fatigued is dangerous and can lead to serious traffic accidents. However, there is a lack of studies on the mechanism of fatigue. This paper sought to infer changes in the cardiovascular system through hand and head skin temperature peripheral factors via an integrated lumped parameter model. A multi-layer inner structure with variable blood perfusion was used to construct a full-body thermal model. The cardiovascular system model provided blood perfusion using lumped parameters. The peripheral resistance and heart rate in the cardiovascular system model were adjusted to match the experimental temperatures of the head and hands obtained from induced fatigue experiments. The simulation results showed that the heart rate and blood pressure decreased, and the peripheral skin resistance of the hands and head increased after fatigue. A decrease in heart rate and an increase in peripheral resistance affect the magnitude of blood flow to the periphery of the body, leading to a decrease in skin temperature during fatigue. The present integrated model elucidates a key effect of human fatigue on the cardiovascular system, which is expected to help improve the accuracy of fatigue monitoring systems.

## 1. Introduction

According to the State Statistics Bureau, there were 2.37 million traffic accidents nationwide between 2012 and 2020, 21% of which were caused by fatigue. Therefore, it is crucial to study fatigue driving detection methods. Driver fatigue monitoring systems have been extensively studied. Domestic and international efforts to monitor driving fatigue are characterized by two main aspects: driving behavior and physiological features. Driver fatigue monitoring systems detect driver fatigue using behavioral features such as the driver’s speed [1] and steering wheel motion [2]. However, the recognition accuracy is not high because of the influence of vehicle type, road conditions, and personal driving habits. Therefore, many researchers have studied driver fatigue monitoring systems using physiological signals such as electroencephalogram [3], electrooculography, electrocardiography, and eye and mouth movements. Although the accuracy of these physiological signals for fatigue monitoring is high, most physiological data measurements are intrusive, and the signals are challenging to extract. A suitable solution is to use less intrusive and more easily extractable physiological signals as auxiliary markers to monitor fatigue.

As an important physiological index of the human body, the temperature can be obtained through non-contact to monitor fatigue. Theoretical studies have found that driving fatigue is closely related to arousal level, which decreases when the driver is fatigued. Researchers [4,5] have found that the temperature of parts of the human body, such as the nose and fingers, can reflect the activity of the autonomic nervous system and be used to track arousal levels. Because the autoregulatory system regulates the cardiovascular system, including heart rate (HR) and the peripheral vasomotor system, skin temperature changes occur in the fatigue state. Moreover, in Jagannath’s [6] simulated driving fatigue test, subjects were found to have altered HR and blood pressures before and after fatigue.

To obtain skin temperature, it is necessary to build a biological heat transfer model. Mathematical heat transfer models of biological systems have been the subject of extensive research by various biologists, physicians, mathematicians, and engineers. Many bioheat transfer models have been used to analyze heat transfer in skin tissue in previous studies, and the bioheat transfer model of Pennes is the most widely used model for studying temperature distribution. In 1948, Pennes presented a solution for biological heat transfer in the forearm. Wissler [7], Wyndham and Atkins [8] extended the model to the entire body. Multi-segment models [9,10,11,12,13] usually include explicit simulations of heat transport and thermal regulation processes in the human body, considering characteristics such as body area, heat capacity, and tissue stratification [14]. Tanabe et al. [10] modeled the heat transfer process of biological tissues in layers, providing separate heat transfer parameters and establishing energy balance equations for different tissue layers. However, Tanabe’s blood perfusion model depends on local skin and core temperatures. With the continuous development of cardiovascular modeling, a cardiovascular system (CVS) model with lumped parameters [11,13] has been used to improve the accuracy of tissue blood perfusion.

Human heat transfer models have been widely used; however, most of these approaches [15,16] have only used empirical models to assess blood flow and heat transfer from the body core to the body surface without considering the real closed-loop cardiovascular system. Blood flow is an essential factor that influences body heat diffusion and skin temperature, and external conditions can affect skin microcirculation and lead to changes in skin temperature [17,18]. Owing to the complexity of cardiovascular networks, the lumped parameter method is the best choice for building a CVS model due to its simplicity of modeling, ease of solution, and low computational effort. The lumped parameter model (LPM) of cardiovascular systems can be used not only to study the hemodynamics of the cardiovascular system but also to explain the link between hemodynamics and heat transfer in the human body [12,19].

In this study, an integrated full-body thermal model based on lumped parameters was built to predict the temperature of different body parts and research changes in the cardiovascular system during fatigue. Infrared thermography temperature measurements were conducted before and after fatigue in eight young adults using fatigue induction experiments. The hand and face temperatures of subjects under normal waking conditions were used to deduce tissue layer thickness and skin resistance. Changes in different subjects’ heart rates and skin resistance were inversely evaluated using the post-fatigue hand and face temperatures considering the skin characteristics of each subject. Finally, changes in the cardiovascular system before and after fatigue were compared.

## 2. Materials and Methods

**Figure 1:**
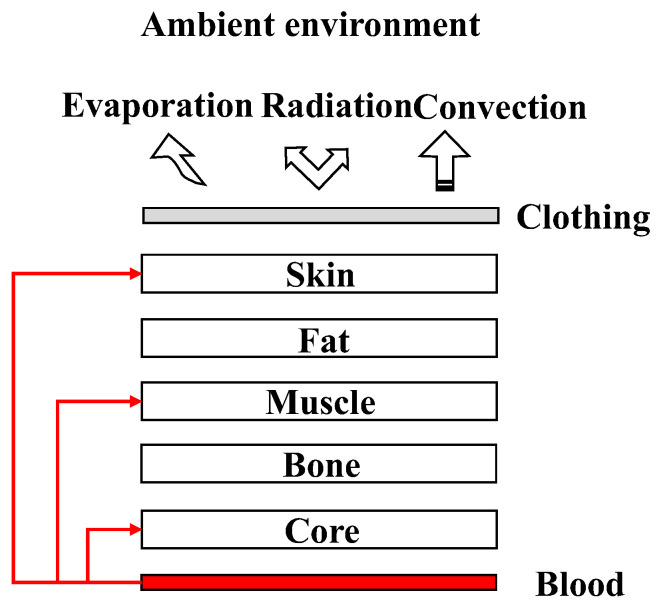
Conceptual figure of the integrated model.

The primary objective of this study was to construct a simulated model of flow heat transfer in the human body using an LPM. In contrast to previously published studies, the cardiovascular system was considered in the tissue heat transfer model to provide blood perfusion. In this study, a full-body thermal model with CVS was developed. The entire body was divided into four segments (the head, torso, upper limbs, and lower limbs). Owing to the varied tissue composition in different body parts, the head and torso segments were divided into five layers (core, bones, muscle, fat, and skin), and the upper and lower limbs segments were divided into four layers (bones, muscle, fat, and skin). A conceptual illustration of the integrated model is shown in Figure 1. Heat is exchanged between the body and the environment by convection, radiation, and evaporation. Individual segments exchanged heat through conduction. Blood perfusion was distributed in different proportions across various tissue layers.

### 2.1. Heat Transfer Tissue Model

The relationship between skin temperature and local blood perfusion has been an interesting topic for researchers. Due to the presence of different structures and distinct physical properties of each tissue layer, it is challenging to study the thermal response of living skin tissues. For anisotropic tissue, Anders et al. [20] explored the relationship between two layers of skin and blood perfusion using an electrical analog model, which showed the thermal response in human skin.

#### 2.1.1. LPM Model of the Heat Transfer in Tissue

**Figure 2:**
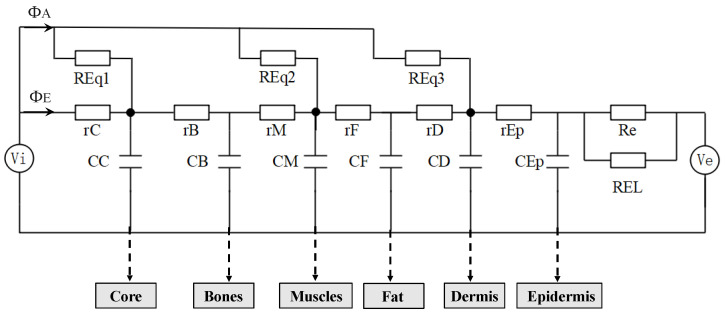
Electrical analog model for head tissues.

Considering tissue thickness, surface area, and physical properties, the process of heat transfer in tissues was simulated using circuit elements. To simplify the calculation, each tissue layer was considered as the same material with effective properties (specific heat *c*, thermal conductance *λ*, tissue density *ρ*, and tissue thickness *l*). The properties of each layer are listed in Table 1 [21]. As shown in Figure 2, the voltage can be represented as an electrical analog measure of temperature. In this model, each layer consists of a serial connection of two elements, each of which is constituted by heat resistance *r* = *l*/*λ* and heat capacity component *C* = *cρl*. *l* represents tissue thickness, and the blood perfusion, *q* ml/cm^2^, generates the heat flux, *Φ*_A_ = *qρc*(*T*_b_ − *T*_i_) W/cm^2^. From this expression, the perfusion thermal resistance can be defined as *R*_Eq_ = 1/*qρc*. Moreover, *V*_i_ simulates the arterial temperature, and *V*_e_ simulates the environmental air temperature. The heat resistance, Re, and REL account for inevitable heat loss during heat isolation and air thermal resistance on the skin surface, respectively. The model parameters are listed in Table 1.

#### 2.1.2. Heat Transfer by Blood Perfusion

It is necessary to determine the blood perfusion of the tissue layer to evaluate heat exchange between tissues and blood. Because the cardiovascular system model can only determine total blood perfusion, the blood perfusion of each tissue layer (BP) must be assigned. In this study, BP was determined by summarizing the relative ratio of blood perfusion of each tissue to total blood perfusion reported in previous research [21]. Thus, the ratio of the blood perfusion of each tissue to the total blood perfusion was calculated using a weighting method.

(1) $$\delta_{ij}=\frac{{BP}_{ij}}{\sum_{j=1}^{m}{BP}_{ij}}$$

where *i* is the number of body segments, including the head, torso, and upper and lower limbs; *m* is the number of tissues; and *j* is the number of tissue layers, including the skin, fat, muscle, bone, and core. 

Total perfusion was distributed according to the proportion of blood perfusion in each tissue layer, as described above. The blood perfusion in each tissue layer was calculated by *ω*_ij_ = *ẟ_ij_*·*q*_i_, where *q*_i_ represents the total flow through the arterioles of the tissue. *ẟ_ij_* of each part is shown in Table 2. The tissue layer innermost to the muscle layer is specified as the core tissue layer, whereas the fat and dermis are skin tissue layers. The calculated blood perfusion is used to calculate the core and skin blood flow resistance and considered core resistance and skin resistance, respectively. Blood flow through these resistances is referred to as core blood flow (CBF) and skin blood flow (SBF), respectively. The specific resistance values are shown in Table 3.

#### 2.1.3. Environmental Resistance

Heat transfer between the environment and body surfaces involves conduction, convection, and radiation. Φ (W/cm^2^) is the heat exchange rate and is described by Equation (2). *h*_t_ (W/cm^2^ °C) is the total heat transfer coefficient from the skin surface to the environment and is expressed by Equation (3). Tsk and Ta represent skin and ambient environment temperatures, respectively. *h*_r_ and *h*_c_ are the convective and radiant heat transfer coefficients, respectively. The environmental resistance was defined as REL = 1/*h*_t_.

(2) $$\Phi =h_{t}(T_{\mathrm{sk}}-T_{a})$$

(3) $$h_{t}=\frac{1}{0.155clo}+h_{r}+h_{c}$$

### 2.2. Cardiovascular System Model

The cardiovascular system (CVS) facilitates the blood perfusion required by the human body and helps maintain a normal skin temperature. As skin temperature and blood perfusion are closely related, modeling the cardiovascular system is beneficial for understanding the mechanism between heat transfer and the cardiovascular system. Hemodynamics in the cardiovascular system can be represented by a set of electrical elements based on the Windkessel model theory, considering the sophistication of the cardiovascular networks. 

Yang’s left heart model [22] was modified to predict blood flow in the entire human body. To obtain arteriolar flow in various parts of the body, blood circulation at the end of the body was enriched based on Liang’s model [23]. In this model, the CVS was divided into six circulation types (heart, cerebral, upper limbs, torso, lower limbs, and peripheral), where the torso and lower circulations were simplified. The CVS model was built using the parameters *R*, *L*, and *C*, where *R* represents blood viscous resistance, *L* represents blood inertia, and *C* represents vessel wall elasticity. These values can be determined based on the anatomy of the vascular segments.

(4) $$R=\frac{8\mu l}{\pi r^{4}},L=\frac{\rho l}{\pi r^{2}},C=\frac{3\pi r^{3}l}{2Eh}$$

where *μ* and *ρ* represent blood viscosity and density, respectively; *E* and *h* denote vascular wall elasticity and thickness, respectively; and *l* and *r* express vascular length and diameter, respectively. 

**Figure 3:**
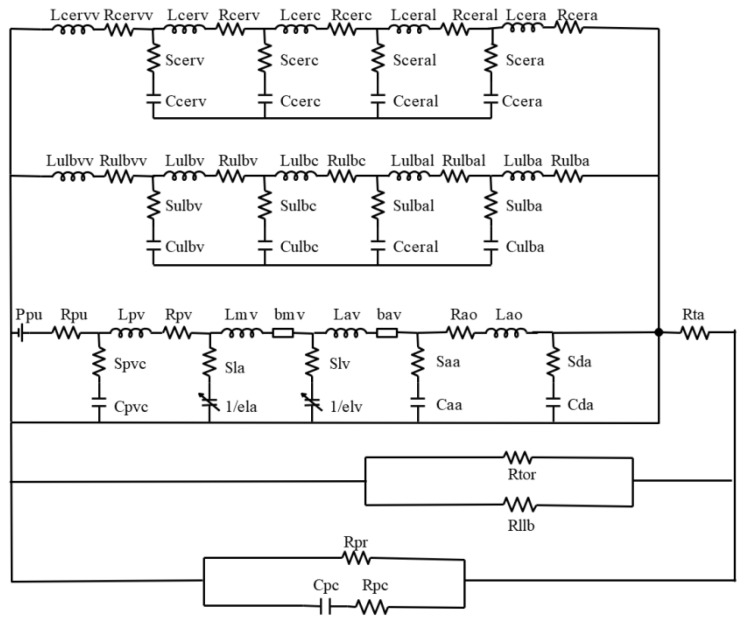
Electrical analogy circuit of the human cardiovascular system.

A complex electrical analog circuit was constructed for the CVS, as illustrated in Figure 3. The model begins with a pressure source P_pu_, which refers to a constant perfusion pressure in the pulmonary capillaries. Pulmonary capillary and venous compliances are lumped into a single capacitance C_pvc_, which fills the left atrium (la) via L_pv_ and R_pv_. The preload of the left ventricle (lv) comes through the mitral valve (mv). The afterload of lv consists of a lumped-parameter representation for ascending aorta (aa), the upper limb circulation (ulb), the cerebral circulation (cer), the thoracic aorta (ta), and descending aorta (da). Meanwhile, blood flows through the thoracic aorta to the lower limbs circulation (llb), including torso circulation (tor) and peripheral vascular circulation (pc). The blood flow in each vessel compartment of the upper limb circulation and cerebral circulation is in analogy with resistance, capacitance and inductance of an electric circuit in series, where R, S, C and L represent blood flow resistance, viscous part of the vessel, compliance of the vessel and blood inertia respectively. The additional subscripts of *a*, *al*, *c*, *v*, and *vv* denote different vessels, including arteries (a), arterioles (al), capillaries (c), veins (v), and venous beds (vv). The RCL parameters were initially determined based on vascular anatomy. In the later stages, the RCL values were partially adjusted by matching blood flow and blood pressure from the relevant literature. The final RCL values are listed in Table 4. As the subcutaneous vascular network is highly complex, the tissue blood perfusion rate is controlled by subcutaneous arterioles. The arterial flow from the cardiovascular system is distributed to the corresponding skin tissue to enable the coupling of the cardiovascular system and skin tissue heat transfer.

### 2.3. Coupled Computation

**Figure 4:**
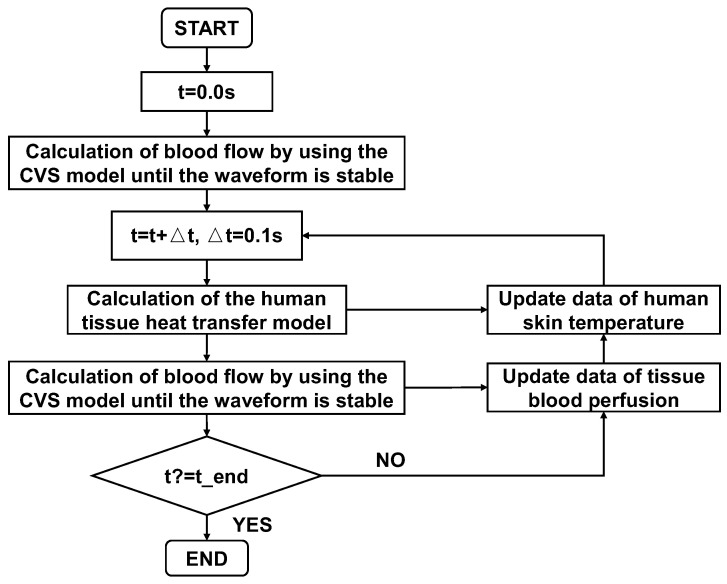
Flowchart of coupling strategy.

In this paper, the CVS model provides the blood flow information for the 0D human tissue heat transfer model to calculate the tissue temperature. The 0D cardiovascular blood circulation model was coupled to the human tissue heat transfer model through the thermal resistance of blood perfusion (*R*_Eq_). The specific computational process is shown in Figure 4.

### 2.4. Experimental Setup

**Figure 5:**
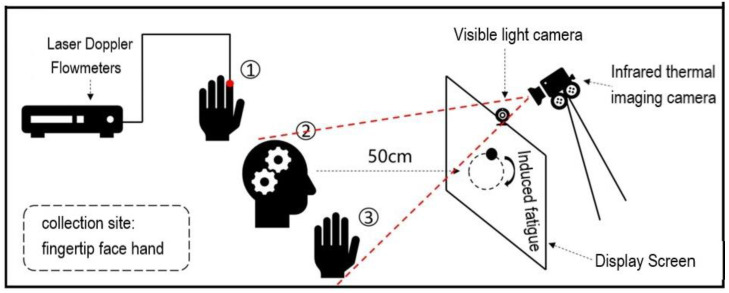
Schematic of the experimental setup for physiological measurement during fatigue state.

Figure 5 shows a schematic of the experimental system for measuring physiological signals such as skin temperature and blood perfusion signals. In this study, fatigue experiments were conducted using infrared thermal imager temperature measurement methods. An infrared thermal imager was used to collect the infrared thermal images of the face and a single hand in the same frame as the subject (Figure 5). A laser Doppler flowmeter (AD Instruments) was used to monitor blood perfusion in the index finger. Eight men volunteered to participate in this study. Their mean age, weight, and height were 24 ± 1.1 years, 73 ± 10.5 kg, and 1.7 ± 0.07 m, respectively. The experimental procedure was strictly followed for all subjects to ensure that the data could be compared without interference. All experimental procedures were performed in a controlled laboratory environment.

A small circularly-moving ball was used as the induction source in the infrared thermographic temperature measurement experiment. As the experiment lasted for only 10–15 min, the self-measurement method interfered with the fatigue experienced by the subject. Experimental videos were recorded, and the evaluator assessed the fatigue level of a subject based on the subject’s sleepiness, referring to the NEDO method [24]. The NEDO method is a fatigue-level evaluation criterion based on facial expressions. The fatigue evaluation criteria divided the fatigue into five levels, as listed in Table 5. In this study, the state of the subjects was evaluated by the raters every 30 s. To ensure that the evaluation results were not affected by individual differences among raters, the assessment results of the three raters were averaged to obtain final state evaluation results for each subject. Levels 1–2 were classified as awake, and levels 3–5 were classified as fatigued.

## 3. Data Processing

Infrared thermal imaging cameras only obtain image and temperature matrix information; therefore, it is necessary to extract temperature information from the desired part of the thermal image. The low resolution of the thermal imaging camera and blurred edges of the image result in poor target tracking. The adopted method combines visible-light images with target-tracking methods using computer vision to extract the temperature of the region of interest in the thermal image. The specific methods are as follows:

Step Face-and-hand feature point recognition and region of interest localization for visible images

**Figure 6:**
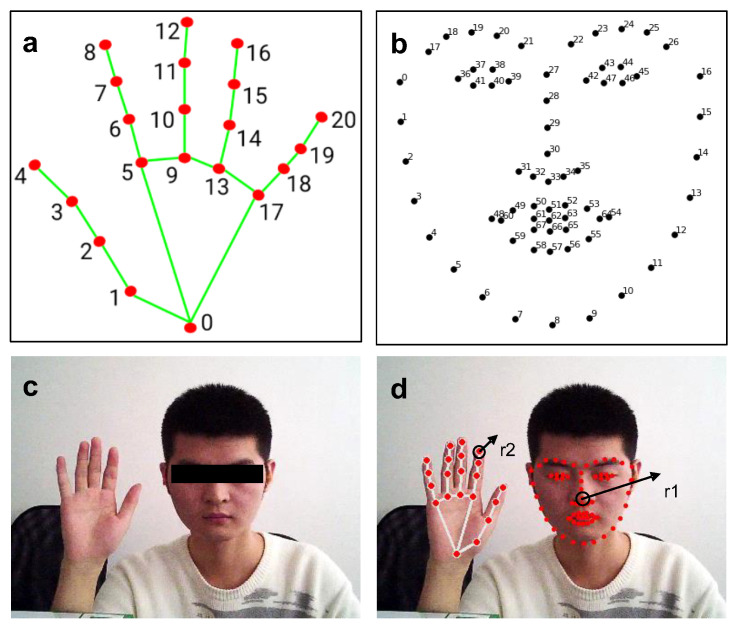
Visible image feature point extraction: (**a**) Schematic of hand 2D feature point distribution identified by Mediapipe open-source library. (**b**) Schematic of facial 2D feature point distribution identified by Dlib open-source library. (**c**) Original image before and (**d**) after feature point identification and region of interest localization.

This paper uses the open-source library Dlib to perform facial feature point recognition on visible face maps. The open-source library uses the HOG method to identify face position recognition in the input vector, calls the already trained facial feature point detector to identify 68 facial feature points (see Figure 6b), and outputs the position coordinates of the feature points. The identified feature points are named from minimum to maximum as f_0, f_1, …, f_67, then f_30 is selected as the center of the tip of the nose, and r1 is the rounded area with a radius of r1, which requires the dynamic calibration of r1 in each frame.

In this study, the open-source library Mediapipe was used for hand-feature point recognition on visible light maps. Similar to Dlib, an open-source library used for facial recognition, the palm region is detected first. Then, a trained hand feature point detector is invoked to recognize 21 hand feature points (see Figure 6a), and the location coordinates of the feature points are output. The identified feature points are denoted as h_0, h_1, …, and h_20, from minimum to maximum. Next, a circular region of radius r2 with h_8 as the center is used as the index fingertip region, where r2 is dynamically calibrated again in each frame.

The first step combined the visible map (Figure 6c) with Dlib and Mediapipe to extract the feature points traced to the region of interest (Figure 6d).

Step Alignment of visible and infrared thermograms

As shown in the previous step, the feature points of the face and hand in the visible image were recognized using Dlib and Mdiapipe, respectively, and the position coordinate sequences of the feature points in the visible image were obtained. Then, we aimed to apply the feature points of the visible image to the corresponding positions of the infrared thermal image with high precision. The visible light camera of the binocular infrared camera used in this study was placed directly above the infrared camera; however, the relative positions of the two cameras remained unchanged. Although the positions of the visible and infrared thermal images are not the same, the difference between the two images is slight. The two images correspond to the same location in space based on image alignment. The dimensions of the visible and thermal images were (1440, 1080) and (384, and 288), respectively. To achieve a better alignment effect, the visible light image size was reduced to the same size as that of the thermal image. The relative offsets (dx and dy) in the horizontal and vertical directions of the two images were then considered for alignment. Due to the lens arrangement of the binocular thermal imaging camera used in this study, the orientation offset direction was considered only in the longitudinal direction. To ensure that the selected image offsets were valid for non-specific target distances, the results of image alignment offsets were tested at different distances from the camera. Finally, 0.5 m spacing and (14, 2) pixel image offset were determined as the optimal settings.

Step Automatic tracking and calibration of areas of interest

Precise mapping of the visible feature points onto the corresponding positions of the thermal image was achieved in Step 2. Then, automatic tracking and calibration of the region of interest were required. As the subject moved to varying degrees during the experiment, the field of view of the face and hand presented in the image changed, which caused the face and hand area in the entire image to change, thus changing the pixel distances r1 and r2 that determine the region of interest. Therefore, it was necessary to determine the field of view change rates for the two frames before and after imaging. When the field of view changes, the distance between adjacent even feature points also changes; therefore, the absolute distance ratio between two points within the current frame and the previous image was calculated using facial feature points f_27 and f_30 as the field-of-view change rate to adjust the size of the region of radii r1 and r2 to improve region localization.

Step Extraction of regions of interest

**Figure 7:**
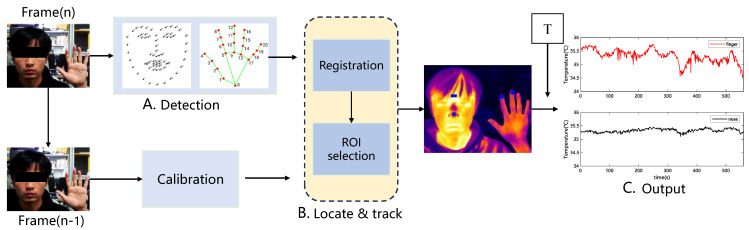
Flow chart of temperature extraction based on feature points in the region of interest.

The final step involved calculating the average temperature inside the localized region of interest. After localizing the thermal image region of interest, the average temperature of the region of interest was obtained by loading a CSV file of the temperature matrix extracted using the software with a thermal imaging camera. Figure 7 shows the algorithm flow for skin temperature extraction within the region of interest based on visible image-assisted thermal images.

## 4. Model Validation

To verify the reliability of the model, the results of the integrated thermal and CVS models were compared with previously published data.

### 4.1. CVS Model and Integrated Model Validation

**Figure 8:**
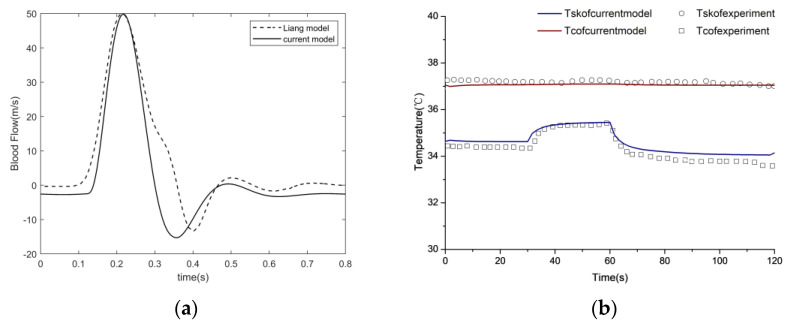
Blood flow wave in the large arteries of upper limbs. (**a**) Simulated and experimental mean skin temperature of the whole body under varying environmental temperatures. (**b**).

The CVS model was validated by comparison with data from Liang’s cardiovascular system model [23]. Comparisons of blood flow in the large arteries of the upper limbs are shown (Figure 8a). The fluctuation in the flow wave from the simulation model in this study was small, which may be related to the compliance parameters of the upper limb arteries. However, good agreement was observed between shape, phase lag, and diastolic reverse flow. The integrated model was validated by comparing the simulated mean skin and core temperatures with published experimental data at various ambient temperatures [13]. As shown in Figure 8b, the simulation parameters were modified based on the environmental conditions used in the experiment. Close agreement was observed between the simulated skin and core temperature and experimental conditions (within 0.5 °C).

### 4.2. Experimental Data Processing and Matching Method for Temperature

**Figure 9:**
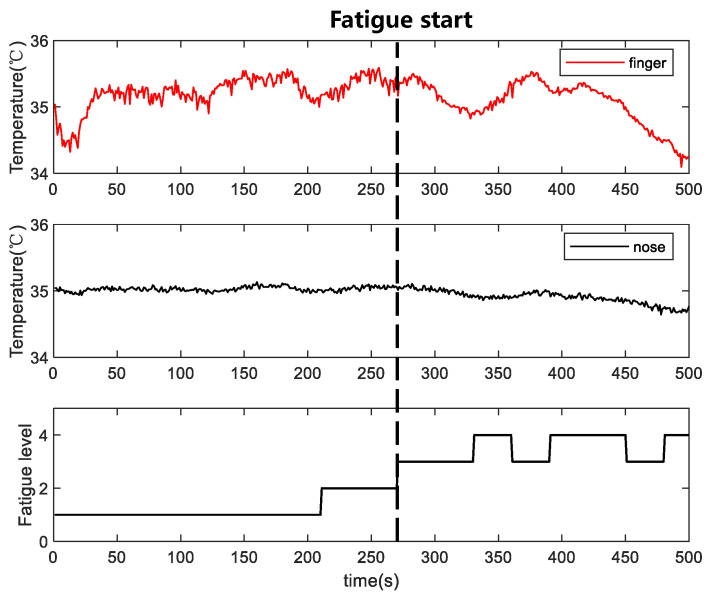
Subject fatigue levels.

The thickness of the skin tissue layer and the skin blood flow differ in each individual. This proposes an individualized model that considers the physiological differences between individuals and the role of blood perfusion in autoregulation. The simulated hand and head temperatures obtained by varying the tissue layer thickness and small skin resistance of the head and hand in the model were used to match the subject’s temperature when awake in the fatigue experiment to obtain a personalized model. The experimental videos of the induced fatigue experiment were used to determine the fatigue level of the participants. In this study, fatigue onset was considered to occur when subjects reached Level 3 fatigue. The average temperature (before fatigue) was considered the temperature when the subject was awake, and the fatigue temperature was considered the average temperature during the Level 5 fatigue state. As shown in Figure 9, the subject became progressively more fatigued and reached a fatigued state at 269 s.

After obtaining skin temperatures before fatigue, an integrated model was developed to vary tissue layer thickness and skin resistance to match the subject’s temperature when awake during the experiment. The changes in tissue layer in heat transfer models reflect differences in physiological structure, and the changes in skin resistance reflect the role of blood perfusion in thermoregulation. An objective function was constructed by calculating the difference between the simulated and experimental temperatures, and the interior-point method was used to solve the constrained optimization problem. The matlabRa2020 version was used to solve the optimization problem. After obtaining the tissue layers and skin resistance for all subjects, a tissue heat transfer model with the CVS model was developed to vary HR and skin resistance to match temperatures during fatigue.

## 5. Results

### 5.1. Skin Temperature Changes after Fatigue Onset

**Figure 10:**
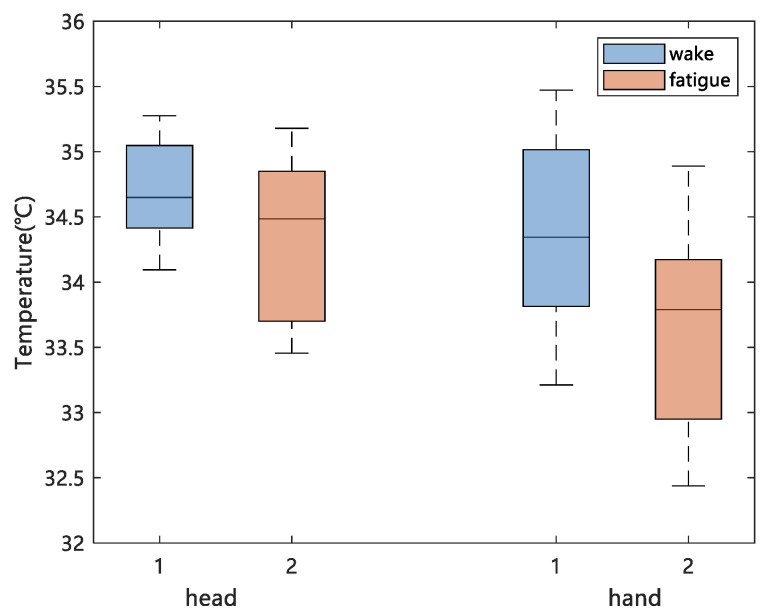
Box plots of pre- and post-fatigue changes in head and hand skin temperature.

During the induced fatigue experiment, skin temperatures of the hand and head regions of interest were collected from eight subjects. As the fatigue level increased, the temperatures of the heads and hands of the subjects gradually decreased, as shown in Figure 9. During the experiment, the skin temperature fluctuation of the hand was large compared to the skin temperature fluctuation of the head. The statistical results of the skin temperatures before and after fatigue in subjects’ hands and heads are shown in Figure 10. The method for calculating the average temperature before and after fatigue is presented in Section 4.2. Comparing the average temperature among the eight subjects before and after fatigue, the average temperature in the head and hand decreased to 0.36 °C and 0.73 °C, respectively, indicating that the mean temperature in the hand decreased more after fatigue.

### 5.2. Physiological Differences between Individuals

**Figure 11:**
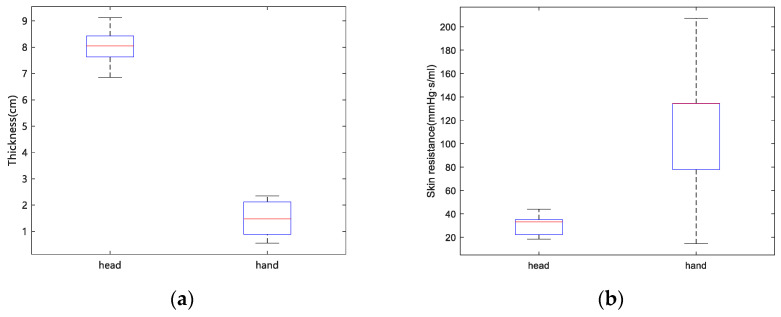
Box plots of tissue layer thickness (**a**) and skin resistance (**b**) of hand and head.

Differences in human skin tissue and blood perfusion can lead to differences in skin temperature between individuals. To investigate the effects of skin tissue thickness and blood perfusion rate on epidermal temperature, the tissue layer thickness and blood perfusion rate of each subject were matched. In this model, the skin resistance and skin blood perfusion rate are associated: when skin resistance increases, the skin blood perfusion rate decreases. Skin temperature is influenced not only by the thickness of the tissue layer and skin resistance (blood perfusion rate) but also by the ambient temperature. Therefore, ambient temperature during the experiment was considered. Using the matching method described in Section 4.2, the thickness of each tissue layer and the magnitude of skin resistance was obtained for all subjects (Figure 11). From the results, it was observed that for different subjects, the skin layer thickness and skin resistance were different. The hand tissue layer thickness was smaller than that of the head, with average tissue layer thicknesses of 1.5 cm and 8.0 cm, respectively. In contrast, the skin resistance of the head was smaller than that of the hand, with average resistance values of 30.4 mmHg·s/mL and 114.4 mmHg·s/mL, respectively. This difference was attributed to higher blood perfusion in the head than in the hand under normal conditions. 

### 5.3. Change in the Cardiovascular System after and before Fatigue

**Figure 12:**
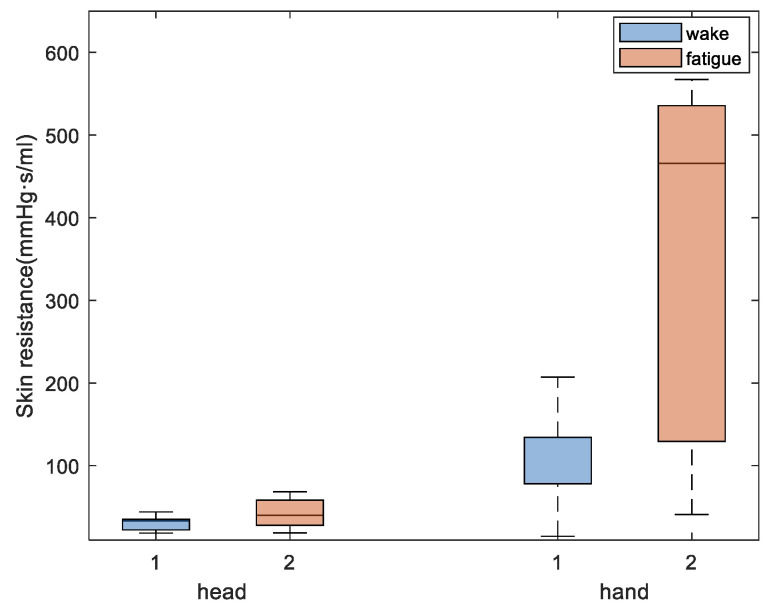
Box plot of skin resistance before and after fatigue.

During the fatigue experiments, we collected head and hand temperature signals from the subjects according to the fatigue rating evaluation described in Section 4.2 (In Figure 9). Based on the specific tissue layer thickness of each subject used in the integrated model, the temperature values of the subject after fatigue during the experiment were matched by varying HR and skin resistance. The initial value of the optimization problem was set to 76.6 beats/min for HR, and the initial value of skin resistance was the skin resistance of the subject in the awake state. Taking the subject in Figure 9 as an example, the head and hand temperatures before fatigue were 35.03 and 35.22 °C, respectively; the skin temperatures of the head and hand after fatigue were 34.77 and 34.25 °C, respectively; and the initial values of the model head and hand were set to 24.55 and 21.96 mmHg·s/mL, respectively. By matching the skin temperatures of the head and hand after fatigue (see Section 4.2 for details), the post-fatigue HR, head, and hand skin impedance values were calculated as 75.9 beat/min, 32.67 mmHg·s/mL, and 80.73 mmHg·s/mL, respectively. HR and skin resistance of all subjects before and after fatigue were calculated (Figure 12). The simulated results indicate that after fatigue, HR decreased, and skin resistance increased in all subjects. The mean HR decreased significantly from 76.6 to 67.96 beats/min. The skin resistance of the head increased from 30.4 mmHg·s/mL to 42.41 mmHg·s/mL after fatigue, and the skin resistance of the hand also increased from 114.4 mmHg·s/mL to 358.73 mmHg·s/mL after fatigue.

**Figure 13:**
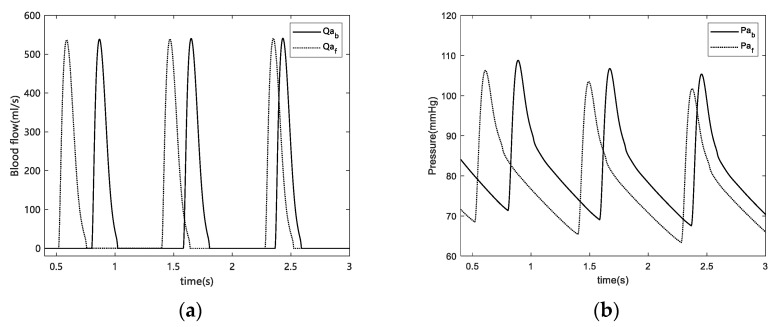
Change of aortic root blood flow (**a**) and blood pressure (**b**) before and after fatigue. Qab is aortic flow in the awake state; Qaf is aortic flow in the fatigued state; Pab is aortic pressure in the awake state; Paf is aortic pressure in the fatigued state.

**Figure 14:**
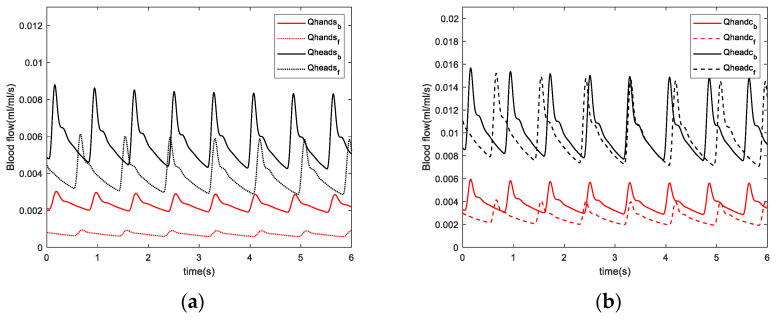
Change of skin blood perfusion (**a**) and core blood perfusion (**b**) before and after fatigue. Qhandsb is hand skin blood flow in the awake state; Qhandsf is hand skin blood flow in the fatigue state; Qheadsb is head skin blood flow in the awake state; Qheadsf is head skin blood flow in the fatigue state; Qhandcb is hand core blood flow in the awake state; Qhandcf is core skin blood flow in the fatigue state; Qheadcb is head core blood flow in the awake state; and Qheadcf is head core blood flow in the fatigue state.

The mean values of HR and skin resistance before and after fatigue were calculated separately for each subject and incorporated into the model to compare changes in the cardiovascular system before and after fatigue. The simulation results revealed a decrease in peak aortic flow (Figure 13a), blood pressure (Figure 13b) and subcutaneous blood perfusion rate in the head and hands (Figure 14) before and after fatigue. The reduction in HR (76.6–67.96 beat/min) and blood pressure (106.6–103.2 mmHg) obtained by the model are consistent with previously reported findings [6]. As HR decreases, aortic flow and blood pressure decrease as well. Although excellent skin resistance in the hands and head leads to an increase in blood pressure, the increase in blood pressure due to the change in skin resistance is not sufficient to counteract the decrease in blood pressure due to the decrease in HR, which was reflected in the simulation results. In this work, it is found that the average blood perfusion of the hand and head skin decreased by 0.0016 mL/mL/s and 0.0018 mL/mL/s, respectively.

## 6. Discussion

Fatigue is a complex physiological phenomenon that affects both physical and mental activity. This study attempted to establish a biological thermal model considering CVS parameters to analyze changes in HR, skin blood flow, and blood pressure in the fatigue stage. It is found that changes in peripheral resistance and HR caused by fatigue are the primary reasons for skin temperature reduction, leading to changes in the cardiovascular system.

Previous studies have shown that the physiological differences between individuals and skin blood perfusion have a significant influence on heat transfer [25,26,27]. Therefore, this paper proposes a method for establishing a personal biological thermal model based on experimental personal data that thoroughly considers differences in tissue layers and skin blood perfusion. Based on the model, some results can be obtained by varying HR and skin resistance. There is something to discuss the simulation results of skin blood perfusion and blood pressure.

For skin blood perfusion, increased peripheral resistance and reduced blood flow induce changes in skin temperature during fatigue. The reduced skin blood perfusion rates in the hands and head after fatigue were consistent with the reduced Laser Doppler blood flow signal components in the fingers identified in the experiment. This suggests that reduced blood perfusion rate after fatigue leads to a decrease in skin temperature, which is consistent with previously reported results obtained from simulated driving experiments [28]. The skin resistance values are expected to change more significantly due to fatigue in the hand than that in the head because skin temperature changes in the hand are strongly influenced by blood perfusion. Moreover, the high oxygen consumption in the head and the stability in blood flow are crucial to maintaining oxygen consumption in the brain, so temperature fluctuations are expected to be less significant in the head.

For blood pressure, we calculated changes in HR and blood pressure after fatigue. HR decreased at fatigue onset, which is consistent with the results of other studies on simulated driving fatigue [6,29]. Regarding the change in blood pressure, a marked decrease in both systolic and diastolic blood pressure was observed before and after fatigue. Our results exhibited consistent trends with Jagannath et al. However, our results contradicted those reported by Fumio [30] and Yamakoshi [28], where the blood pressure increased in their experiments. This was caused by differences in driving environments. In Fumio’s experiment, the subjects studied were city drivers where the urban traffic environment was worse, which results in tremendous stress and can lead to nervousness or anxiety, thus potentially increasing blood pressure. In Yamakoshi’s [28] experiment, the simulated road conditions were highway environments. However, subjects were given clear demands, such as “keep an eye on surroundings“ and asked to perform ongoing monotonous tasks under strained conditions. Therefore, the demand for prolonged attention causes stress in drivers and may increase blood pressure.

Furthermore, the blood pressure response is influenced by local conditions in the working muscles, which are peripherally regulated by a muscle chemical reflex of metabolites in the trapped muscles and remain continuously elevated as long as the occlusion persists [31]. Therefore, during previous experiments, there is a possibility that peripheral regulation by muscle chemical reflexes may be responsible for the increase in blood pressure. However, the present experiment did not exceed 1 h in length, and the subjects were not asked to pay constant attention to their surroundings, which allowed them to reach a state of fatigue in a safe situation. Therefore, the subjects’ blood pressure was not expected to increase in response to tension.

By extension, drivers experience higher stress levels under sustained mental stress. Moreover, as mental fatigue increases, HR decreases, and HR variability (HRV) increases. Increased HRV implies that the autonomic nervous system inhibits vagus nerve activity and increases sympathetic nerve activity, which may be related to the protection of physiological coherence [32]. In addition, during fatigue, excitatory sympathetic activity activates the renin-angiotensin system (RAS), which produces angiotensin that acts on vasoconstriction [33], leading to the contraction of skin capillaries, a decrease in tissue blood perfusion, and a decrease in skin temperature. In future studies, wavelet analysis could be applied to analyze changes in endothelial and neural frequency segments to explore the reasons for the decrease in blood flow due to the increase in peripheral resistance. When peripheral resistance increases, blood pressure increases, and the increase in blood pressure stimulates blood pressure receptors, which can reduce HR. Not only does the blood perfusion rate in the superficial layer decrease, but the decrease in HR also leads to a decrease in blood perfusion in the core layer of the tissue. The effects of fatigue on the cardiovascular system are characterized by a decrease in HR as well as a decrease in blood pressure and a decrease in aortic root flow before and after fatigue, suggesting that HR and blood pressure influence the magnitude of blood flow to the peripheral body [34]. Therefore, in a fatigued state, the blood supply to the brain may be reduced, and the brain requires more oxygen to function correctly. Slight hypoxia may also be a cause of fatigue in the body, and a reduction in saturated blood oxygen to the brain has been reported in previous experiments [35].

Finally, although the experimental results of only 8 subjects are presented in this paper, a lot of work has been done to verify the model. In the early experiments, the thermocouple was used to collect skin temperature signals and obtain data from 13 subjects. Based on these results, we found that hand and head temperature decreased after fatigue, which is consistent with infrared measurements. Since the infrared thermal imager can measure temperature without contact, which is more suitable for our research purposes, we used the infrared thermal imager to capture temperature in the later experiment. And the infrared thermal imager can get the temperature information of the whole face space. In the following work, it is necessary to further expand the experimental data of the infrared thermal imager and improve the reliability of the model.

## 7. Limitations

This study had some limitations. The blood perfusion in each tissue layer was uneven. Although the weighted method was used to assign blood perfusion, it is necessary to collect more samples from each tissue layer using laser Doppler measurements to decrease deviation. In addition, the CVS model was simplified in this work, and the vascular network system of the lower limbs and trunk was only considered using electrical resistance. The results obtained by the model would be more accurate if a more comprehensive systemic cardiovascular system could be established. Moreover, a lumped parameter model was used to build the CVS model, and the parameters of the CVS model were difficult to determine because they were taken based on blood flow waveforms in previous literature. However, changes in the parameters have a significant impact on blood flow. Further studies are required to enhance the accuracy of this model.

## 8. Conclusions

In this study, an integrated biological thermal model with a complex lumped parameter cardiovascular model was built to predict the thermal response of skin tissue after fatigue onset. The temperature during the fatigue state was determined through an induced fatigue experiment. Dynamic skin blood perfusion and individual differences were considered in the model. Skin temperatures of the hand and head were determined before and after fatigue, and it was confirmed that the model effectively predicted the skin temperatures of the hand and head. The mean absolute errors of the proposed model in predicting skin temperatures of the hand and head during fatigue were within acceptable limits. The model was used to predict changes in the cardiovascular system before and after fatigue; it was found that HR decreased, peripheral resistance increased, and both aortic blood flow and blood pressure decreased. HR and peripheral resistance combine to influence the magnitude of blood flow to the periphery of the body, leading to a decrease in skin temperature during fatigue. Finally, the model may help offer a preliminary strategy for predicting the pathological and physiological interactions between skin temperature and blood perfusion and is expected to support the development of new monitoring strategies for driving fatigue.

## Figures and Tables

**Table 1:** Thermophysical properties and model parameters of tissues.

		λ W/m °C	*Ρ* kg/m³	c J/kg °C	r W/cm^2^ °C	C J/cm^2^ °C
Head	Core	0.53	1360	2450	222.64	3.93
	Bond	1.2	1300	1590	14.17	0.35
	Muscle	0.5	1050	3770	76	1.50
	Fat	0.21	850	2500	71.43	1.28
	Dermis	0.37	1200	3400	22.97	1.39
	Epidermis	0.26	1200	3600	3.08	0.035
Torso	Core	0.53	1360	2450	359.18	5.86
	Bond	1.2	1300	1590	11.67	0.29
	Muscle	0.5	1050	3770	236	4.67
	Fat	0.21	850	2500	300	1.28
	Dermis	0.37	1200	3400	32.43	0.49
	Epidermis	0.26	1200	3600	3.08	0.035
Upper limbs	Core	−	−	−	−	−
	Bond	1.16	1300	1590	86.21	2.07
	Muscle	0.5	1050	3770	256	5.07
	Fat	0.21	1000	3060	128.57	0.83
	Dermis	0.37	1200	3400	45.95	0.69
	Epidermis	0.26	1200	3600	3.08	0.035
Lower limbs	Core	−	−	−	−	−
	Bond	1.16	1300	1590	86.21	2.07
	Muscle	0.5	1050	3770	220	4.35
	Fat	0.21	1000	3060	333.33	1.49
	Dermis	0.37	1200	3400	41.89	0.63
	Epidermis	0.26	1200	3600	3.08	0.035

**Table 2:** Tissue layer thickness, blood perfusion, and *ẟ_ij_* of each body part.

	Tissue	*l* (cm)	A (cm^2^)	Volume (cm^3^)	Volumetric Perfusion (mL/s/kg)	Perfusion (mL/s)	*ẟ_ij_* (%)
Head	Core	1.18	1128	1331	9.21	12.97	91
	Muscle	0.38		429	0.55	0.23	1.6
	Fat	0.3		338	0.004	0	0
	Skin	0.17		191	4.9	1.0	7.4
	Bond	0.17		191	0	0	0
	total	2.2		2480	−	14.2	100
Torso	Core	1.76	6016	10,588	4.3	48	90
	Muscle	1.18		7110	0.51	3.81	7.1
	Fat	0.7		4211	0.004	0.018	0.03
	Skin	0.12		722	1.9	1.5	2.87
	Bond	0.14		842	0	0	0
	total	3.62		23,473	−	53.328	100
Upper limbs	Muscle	1.28	3180	4070	0.6	2.52	85.1
	Fat	0.27		858	0.004	0.003	0.1
	Skin	0.1		318	1.3	0.44	14.8
	Bond	0.3		870	0	0	0
	total	1.95		6549	−	2.963	100
Lower limbs	Muscle	1.1	6392	7000	0.51	3.75	75.7
	Fat	0.7		4474	0.004	0.013	0.26
	Skin	0.155		990	1.0	1.1	24.04
	Bond	0.5		3196	0	0	0
	total	2.85		15,660	−	4.95	100
Hand	Muscle	0.3	1000	300	0.25	0.14	17.5
	Fat	0.15		150	0.0075	0.0012	0.15
	Skin	0.2		200	3.3	0.66	82.35
	Bond	1		1000	0	0	0
	Total	1.65		1650	−	0.8	100

**Table 3:** Parameter values for microcirculation resistance.

	Core Resistance (mmHg·s/mL)	Skin Resistance (mmHg·s/mL)
Head	4.7	62
Torso	2	68
Upper limbs	30	189
Lower limbs	28	96

**Table 4:** Cardiovascular system model parameters.

	**Preload**	Ppu(mmHg)	7.4	**Aortic trunk**	Aav (cm^2^)	4.0
		Rpu (mmHg·s/mL)	0.01		Lao (mmHg·s^2^/mL)	0.0008
		Rpv (mmHg·s/mL)	0.002		Lav (mmHg·s^2^/mL)	0.0004
		Lmv (mmHg·s^2^/mL)	0.0005		Caa (ml/mmHg)	0.1
		Lpv (mmHg·s^2^/mL)	0.0005		Cda (ml/mmHg)	0.1
		Amv (cm^2^)	4.0		Rao (mmHg·s/mL)	0.04
					Rta (mmHg·s/mL)	0.02
	**Artery** **(E_0_:Z:R:L)**	**Arteriole** **(C:R:L)**		**Capillary** **(C:R:L)**	**Vein** **(C:R:L)**	
Head	0.6:15:0.04:0.002	0.2:4.3:0.003		0.6:2.09:0.0005	2.3:0.55:0.0004	
Upper limbs	0.8:13:0.1:0.003	0.1:25.89:0.003		0.45:4.18:0.0005	4.6:1.09:0.0004	
Torso	Rtor = 1.94					
Lower limbs	Rllb = 21.68					
Peripheral	Rpr = 1.4	Rpc = 0.01		Cpc = 2.0		

R, mmHg·s/mL; L, mmHg·s^2^/mL; C, ml/mmHg.

**Table 5:** Fatigue rating criteria based on facial expression by the NEDO procedure.

Level	State	Facial Expressions and Behaviors
Level 1	Awake	Eye movements are quick and frequent Blink rates are stable at approximately two per 2 s Body motions are active
Level 2	Slightly drowsy	Lips are parted Motions of eye movements are slow Body motions are active
Level 3	Drowsy	Blinks are slow and frequent Repositions body on the seat Touches hand to face
Level 4	Very drowsy	Blinks are assumed to occur consciously Shakes head Frequently yawns
Level 5	Extremely drowsy	Eyelids closing Moves head back and forth

## Data Availability

Correspondence and requests for materials should be addressed to X.D.
